# Supplementary material for: Targeting CD123 in blastic plasmacytoid dendritic cell neoplasm using allogeneic anti-CD123 CAR T cells
Source: Nat Commun. 2022 Apr 28;13:2228. doi: 10.1038/s41467-022-29669-8 (PMC9051102; doi:10.1038/s41467-022-29669-8)
Supplement: Supplementary file 1 — Supplementary Information [file 41467_2022_29669_MOESM1_ESM.pdf]

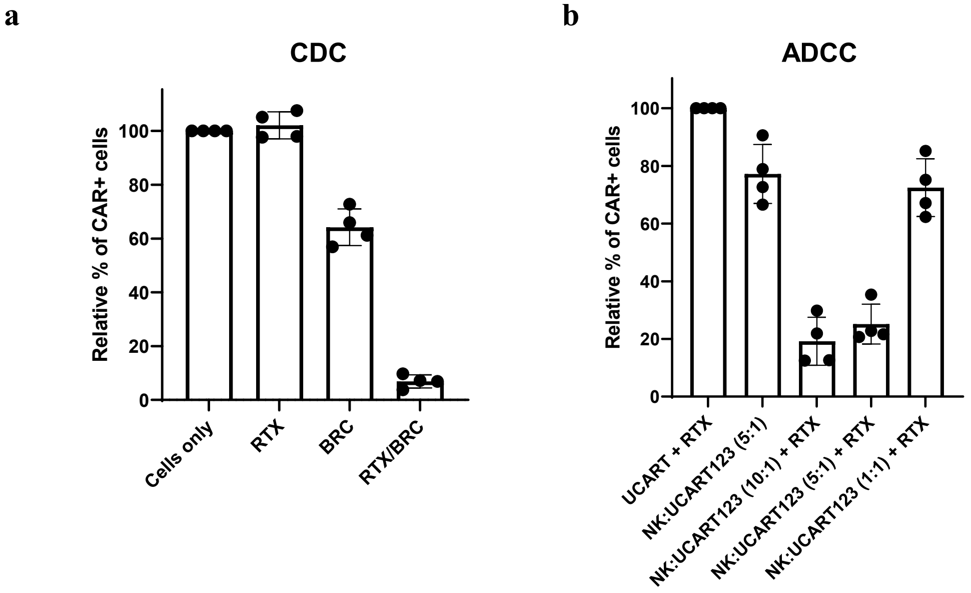

Supplementary Fig. 1. Rituximab mediated depletion of UCART123. Panel **a** shows the relative percentage of CAR+ cells detected after 2h incubation of UCART123 with Rituximab (RTX), baby rabbit complement (BRC) or both, indicating efficient depletion through RQR8 by CDC. Panel **b** shows the efficacy of ADCC when UCART123 cells were co-cultured for 24h with NK cells in the presence or not of RTX at different NK:UCART123 ratios. In both cases, the data presented was obtained using large scale batches of UCART123, in four independent experiments. The mean ± SD value is presented. Source data are provided as a Source data file.

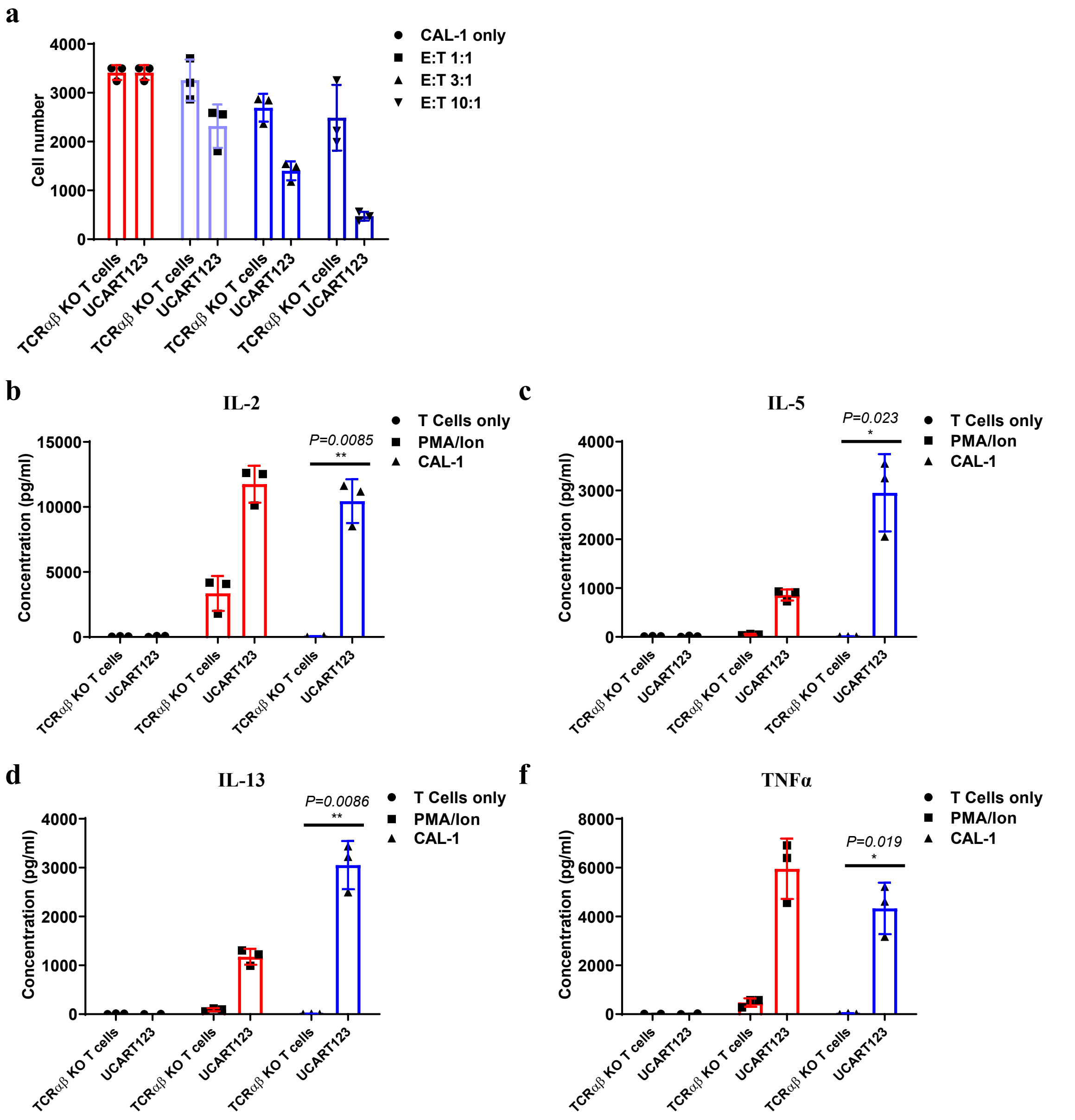

Supplementary Fig. 2. Cytotoxicity of UCART123 against CAL-1 BPDCN cells *in vitro*. a, CAL-1 BPDCN cells were co-cultured with either non-transduced TCRαβ-deficient (TCRαβ KO) T cells or with UCART123 cells at various effector: target (E:T) ratios indicated. After 16 hours co-culture, CAL-1 cell number was quantified and specific cytotoxic activity of UCART123 against CAL-1 target cells was calculated. Each point represents the data obtained from triplicate experiments, and the mean ± SD value is presented. **b-f**, UCART123 or TCRαβ KO T cells were either unstimulated (T Cells only), co-cultured with CAL-1 cells, or stimulated with PMA/ionomycin (PMA/Ion) as a positive control. IL-2, IL-5, IL-13 and TNFα levels were determined by the BioLegend LEGENDplex assay. Data represent n= 3 biological replicates and mean ± SD of triplicates. Significance was determined using unpaired two-tailed t-test annotated as **P≤ 0.05, **P≤ 0.01*. Source data are provided as a Source data file.

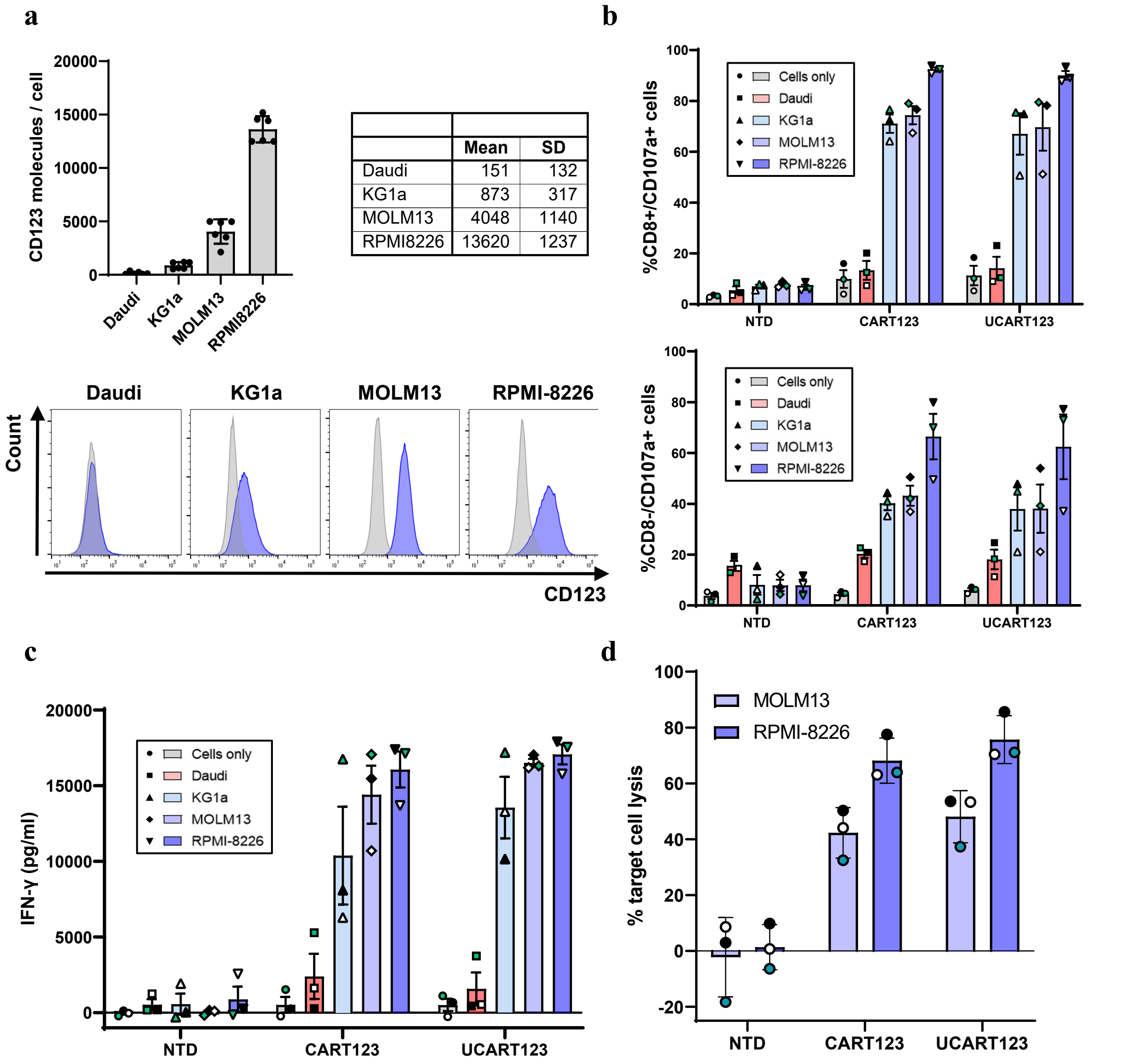

	Mean	SD
Daudi	151	132
KG1a	873	317
MOLM13	4048	1140
RPMI8226	13620	1237

Supplementary Fig. 3. In vitro activity of T-cells expressing CAR123 against cell lines expressing different levels of CD123. a, The upper row of panel a shows the number of CD123 molecules at the cell surface, using the Qifikit surface antigen quantification kit. Daudi cells were used as a CD123 negative control, while KG1a, MOLM13 and RPMI-8226 were used as targets expressing increasing levels of CD123. The table on the upper right portion shows the mean and the SD values from 6 independent staining. Representative flow cytometry data is shown in the lower row of panel a, with blue histograms corresponding to cells labelled with and anti-CD123 monoclonal antibody (clone 6H6), while the grey histograms correspond to cells labelled with the isotype control. **b**, Panel b shows degranulation activity of CAR123 cells, either cultured alone or co-cultured during 6h with the different cell lines described in panel a, at a 1:1 E:T ratio. Degranulation was evaluated by flow cytometry after staining with CD8 and CD107a antibodies. The degranulation of the CD8+ and CD8- fractions (considered as CD4+ cells) is shown. **c**, Panel c shows IFNγ levels released in the supernatants of overnight co-cultures of CAR123 cells with target cells at 1:1 E:T ratio, using an ELISA test. **d**, Cytotoxic activity against MOLM13 and RPMI-8226 cells is shown in panel d. Co-cultures were settled with each of the CD123+ cell lines (together with Daudi CD123neg cells as an internal negative control) at a 10:1 effector to target (E:T) ratio for 18 hours. Cytotoxic activity was evaluated by assessing the viability of the different target cell populations by flow cytometry at the end of the co-culture. Cell killing activity was normalized to the activity against CD123- Daudi cells. Source data are provided as a Source data file.

NTD: non transduced T-cells. UCART123: T-cells transduced with CAR123, transfected with TRAC targeting TALEN® and purified by depletion of remaining TCRαβ+ T-cells. CART123: T-cells transduced with CAR123, mock transfected (TCRαβ+ cells). The experiments shown in panels b-d were performed with T-cells from three different donors, each of which is represented by a different color (black, green or white symbols). The donor highlighted in black was transduced with a R&D backbone, while the two other donors were transduced with an rLV produced from the same backbone used to manufacture UCART123 clinical batches. Nevertheless, all three donors express the CAR from the same EF1αp-RQR8-2A-CAR123 lentiviral expression cassette.

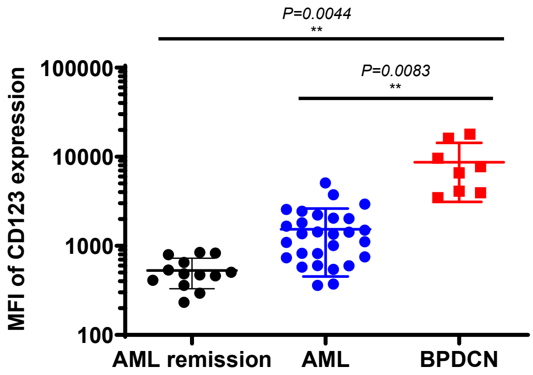

Supplementary Fig. 4. CD123 expression level in BPDCN is higher than in AML. We compared the CD123 level presented as mean fluorescence intensity (MFI) on blasts in bone marrow samples of 13 AML patients in remission, 28 patients with newly diagnosed AML and 8 patients with newly diagnosed BPDCN. CD123 expression levels were significantly higher in BPDCN than in AML remission cells (MFI range 232-845; ***P≤ 0.01*) and AML cells (MFI range 360-5,073; ***P≤ 0.01*). The mean ± SD value is presented. Significance was determined using unpaired two-tailed t-test. Source data are provided as a Source data file.

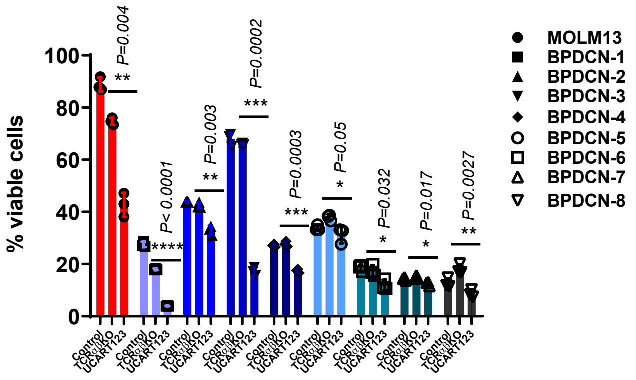

Supplementary Fig. 5. Antitumor activity of UCART123 against primary BPDCN samples in vitro. Viability of MOLM13 cells and of the primary BPDCN samples upon co-culture for 16 hours with either UCART123 cells or non-transduced TCRαβ-deficient (TCRαβ KO) T cells. Target cells from control group were cultured alone in the same experimental conditions as the co-cultures. Each point represents the data obtained from triplicate experiments, and the mean ± SD values are shown. Significance was determined using unpaired two-tailed t-test. **P≤0.05, **P≤0.01, ***P≤0.001, ****P< 0.0001*. Source data are provided as a Source data file.

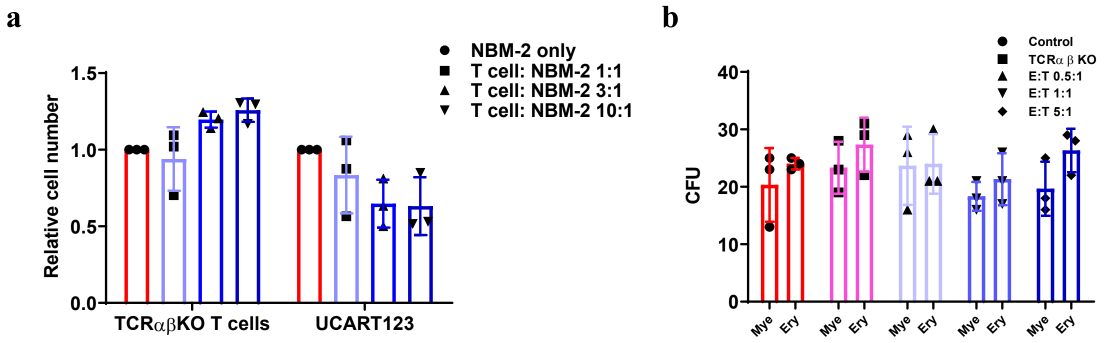

Supplementary Fig. 6. The toxicity of UCART123 against normal hematopoietic cells *in vitro*. a, Normal BM-derived hematopoietic cells were co-cultured with either UCART123 cells or non-transduced TCRαβ-deficient T cells (TCRαβ KO) for 16 hours and counted by flow cytometry. Each point represents the data obtained from triplicate experiments, and the mean ± SD value is shown. **b**, Normal BM-derived hematopoietic stem cells were co-cultured for 2 weeks with UCART123 at different ratios and the cultures analyzed by colony-formation assay. Erythroid colony-forming units (CFUs) and myeloid CFUs were counted separately. Each point represents the data obtained from triplicate experiments, and the mean ± SD value is presented. Source data are provided as a Source data file.

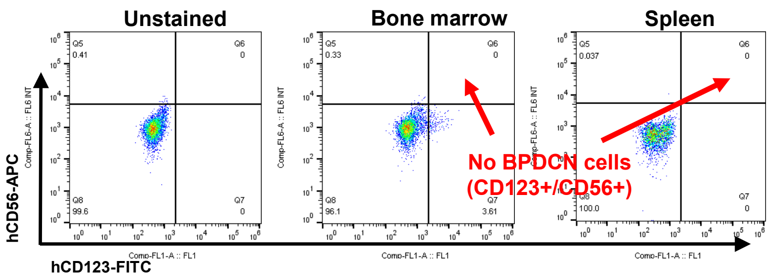

Supplementary Fig. 7. no BPDCN cells (hCD123+ and hCD56+ cells) were detected in bone marrow or spleen of died mouse from 10×10⁶ UCART123 treatment group. Cells were isolated from bone marrow or spleen of UCART123-treated mouse when it died. Expression of hCD123+ and hCD56+ was measured by flow cytometry.

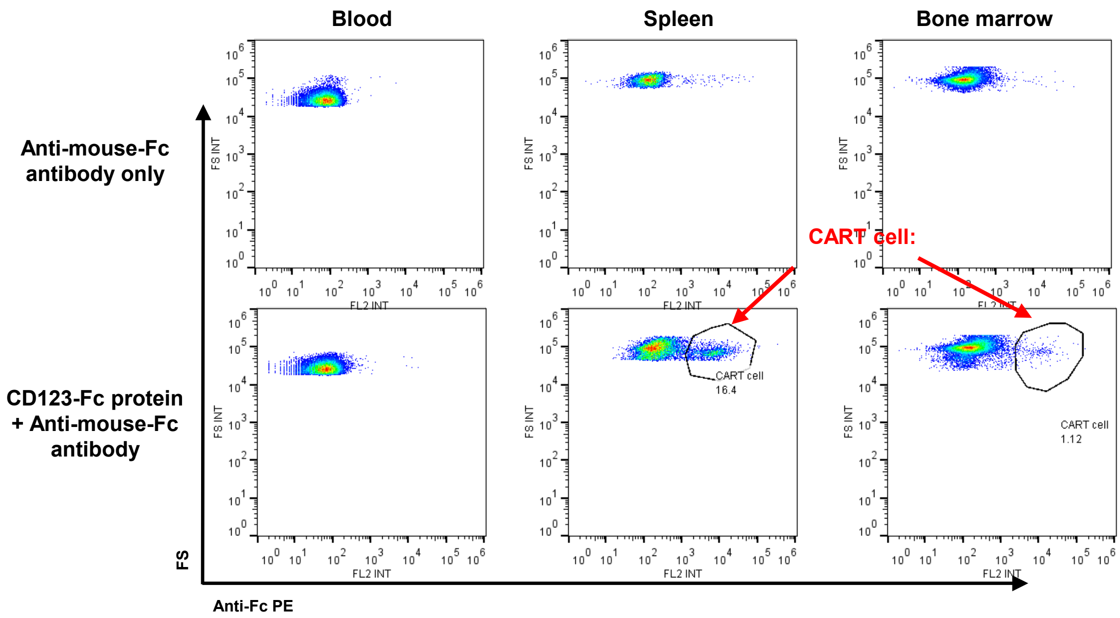

Supplementary Fig. 8. The presence of UCART123 cells was analyzed in the blood, spleen, and bone marrow of the mouse treated with 10×10^6 UCART123 cells (PDX-1 model) and sacrificed on Day 78 (57 days after UCART123 treatment). UCART123 cells were detected by flow cytometry using CD123-Fc protein and an anti-mouse Fc antibody.

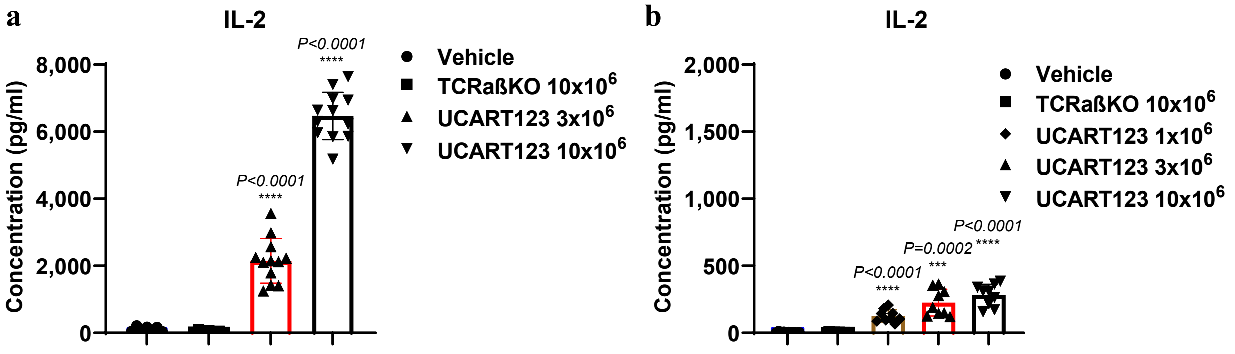

Supplementary Fig. 9. IL-2 levels in peripheral blood were measured 2 days following T cell injection in PDX-2 experiment (a) and repeat PDX-2 experiment (b); a, Experimental design using BPDCN-3 cells. When engraftment was confirmed on day 21 after tumor cell injection (0.1-23.5% circulating BPDCN), mice were randomized into 4 treatment groups and received treatment as follows: vehicle (n=11 mice); 10×10^6 TCRαβ KO T cells (n=11 mice); 3×10^6 UCART123 cells (n=12 mice); or 10×10^6 UCART123 cells (n=12 mice) by single tail vein injection. **b**, Experimental design of a separate experiment with PDX-2 experiment. In this study, mice injected with BPDCN-3 (1×10^6 tumor cells/mouse) received similar treatment, this time in 5 groups (3 UCART123 doses; n=9 mice/group) that was initiated on day 14 after injection of tumor cells, when tumor burden was low (0% circulating BPDCN, 0.9-1.1% engraftment in the bone marrow). UCART123-induced IL-2 release was significantly lower in repeat PDX-2 experiment compared to PDX-2 experiment. The mean ± SD value is presented. Significance was determined using unpaired two-tailed t-test. Comparison to vehicle group: ****P≤ 0.001, ****P< 0.0001.* Source data are provided as a Source data file.

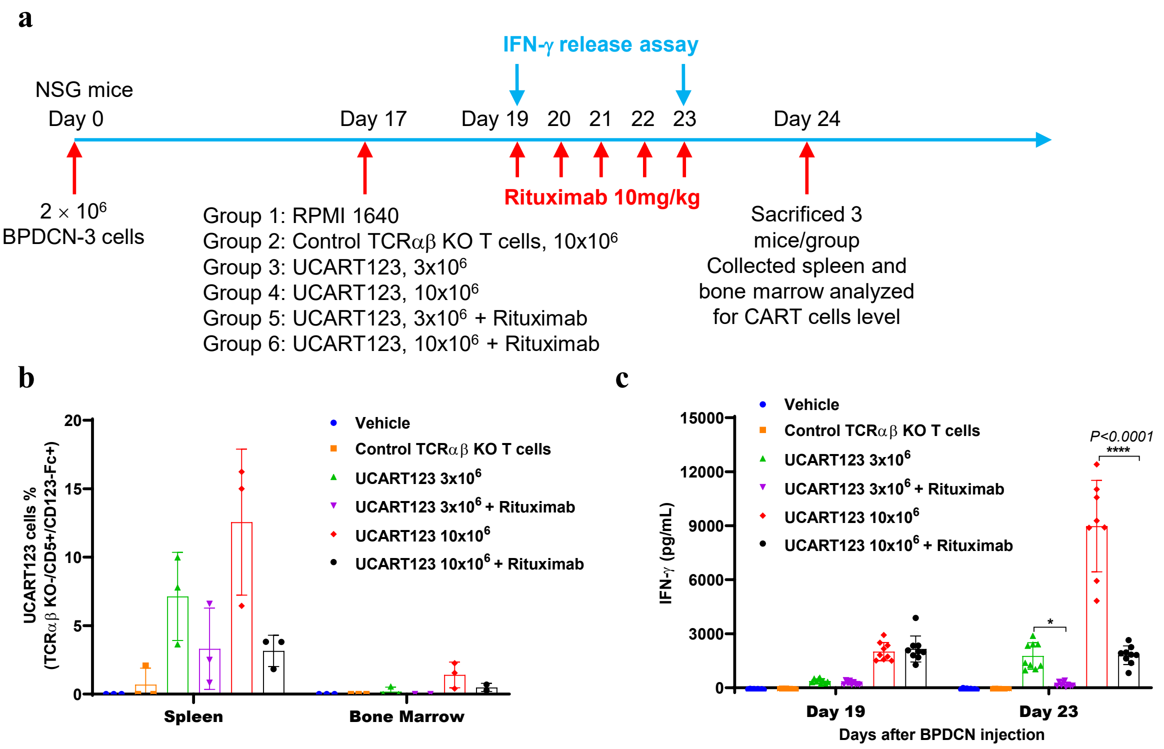

Supplementary Fig. 10. Rituximab depletes UCART123 *in vivo*. **a**, Experimental design using BPDCN-3 PDX cells. When engraftment was confirmed on day 17 after tumor cell injection, mice were randomized into 6 treatment groups (n=9 mice/group) and received treatment as follows: vehicle; 10×10^6 TCRαβ KO T cells; 3×10^6 UCART123 cells; 3×10^6 UCART123 cells followed by Rituximab; 10×10^6 UCART123 cells or 10×10^6 UCART123 cells followed by Rituximab. Rituximab at 10mg/kg was administered i.p. 2 days after UCART injections (day 19) for a total of 5 days. **b**, Fractions of UCART123 cells in the spleen and bone marrow of mouse (n=3) from experimental cohorts. Mice were sacrificed on day 24 after tumor cell injection. UCART123 cells were detected by flow cytometry using CD123-Fc protein and an anti-mouse Fc antibody conjugated with PE. The mean ± SD value is presented. **c**, IFN-γ levels in peripheral blood of mice measured on day 19 and day 23. The mean ± SD value is presented. Significance was determined using unpaired two-tailed t-test. Compared to 10×10^6 UCART123 cells group: ****$P<0.0001$. Source data are provided as a Source data file.

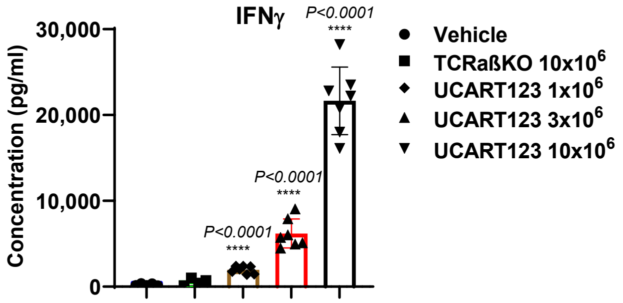

Supplementary Fig. 11. IFNγ levels in peripheral blood were measured 2 days following T cell injection in PDX-3 experiment. UCART123-induced IFNγ release was significantly higher compared to vehicle group. n=9 mice/group and the mean ± SD value is presented. Significance was determined using unpaired two-tailed t-test. ****$P< 0.0001$. Source data are provided as a Source data file.

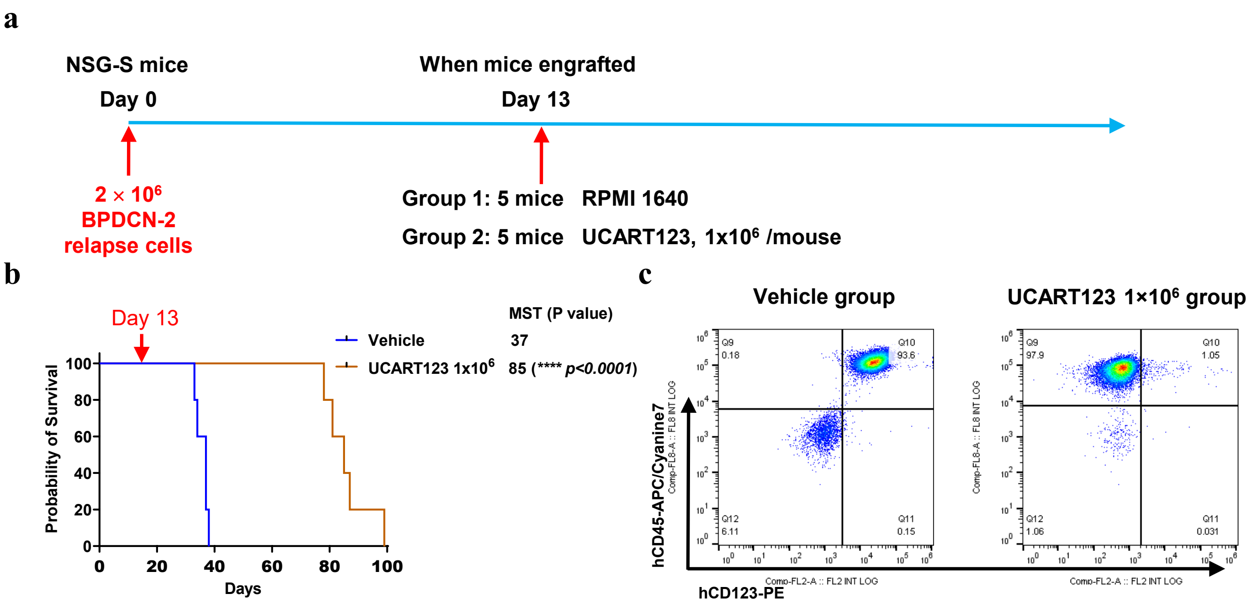

Supplementary Fig. 12. Loss of CD123 leads to escape from UCART123 therapy in a BPDCN relapse experiment. a, To establish a BPDCN relapse experiment, 10 mice were injected intravenously with 2×10⁶ relapse BPDCN tumor cells (CD123+ tumor cells collected from the spleen of a mouse from the group treated with 1×10⁶ UCART123 in the initial PDX-3 experiment). After engraftment on day 13 (0.1-0.2% circulating BPDCN), mice were randomized into 2 treatment groups (n=5 mice/group). Mice received a single intravenous injection of vehicle or 1×10⁶ UCART123 cells. **b**, Survival of mice in the 2 treatment groups was estimated by the Kaplan-Meier method. Red arrow indicates start of treatment (Day 13). Median survival time (MST) are indicated in days. Significance was determined using unpaired two-tailed t-test. Comparison to vehicle group: **** *P<0.0001*. **c**, Expression of CD123 on tumor cells isolated from bone marrow of vehicle-treated or UCART123-treated mice sacrificed due to high tumor burden. CD123 was detected by using CD123-PE and CD45-APC-Cy7 antibodies and gating on viable cells (DAPI$^-$). Similar to initial PDX-3 experiment, tumor cells from UCART123-treated mice lost CD123 (CD123$^-$) but remained CD56$^+$. Source data are provided as a Source data file.

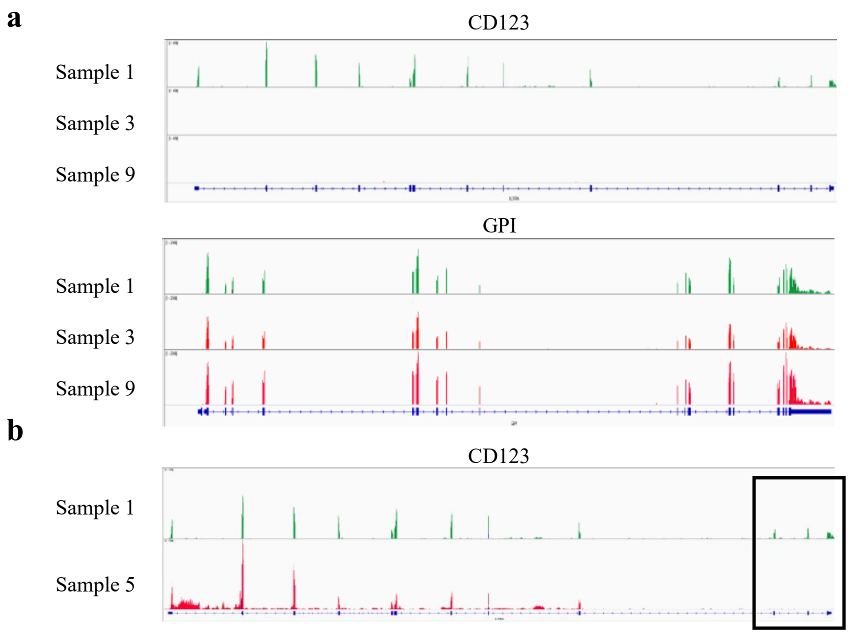

Supplementary Fig. 13. Molecular analysis of CD123 loss during UCART123 treatment. a, RNA-sequencing reads aligned to Genome Browser tracks for CD123 (top) and housekeeping gene GPI (bottom) show no reads present for CD123 but reads present for GPI in samples 3 and 9. **b**, RNA-sequencing reads aligned to Genome Brower tracks for CD123 shows reads spanning the entire exonic region for CD123^{+} samples but a loss of reads at the 3′ end of the gene.

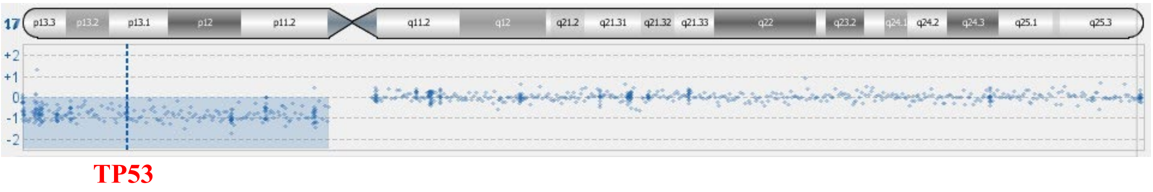

Supplementary Fig. 14. TP53 deletion was detected in the BPDCN-2 patient sample. RNA was isolated from spleen of mouse which was injected with cells from a primary BPDCN sample (from patient BPDCN-2). aGCH analysis showed a large genetic deletion on chromosome 17, compared to sample from normal patient, corresponding to the location of TP53.

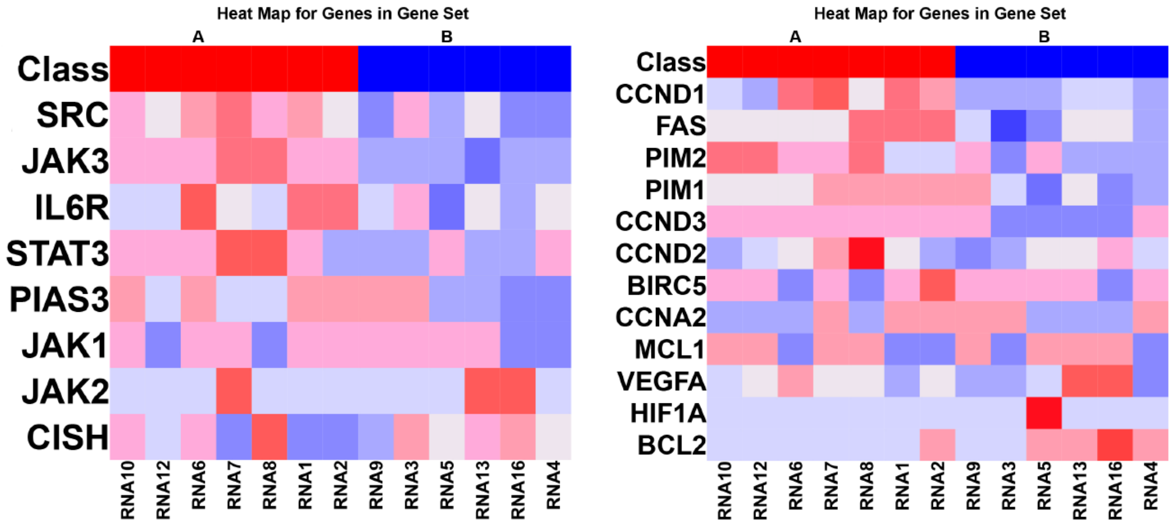

Supplementary Fig. 15. Gene Set Enrichment Analysis. Pathway analysis was carried out using Gene Set Enrichment Analysis (GSEA) method. Group A is CD123+ samples and group B is CD123- samples.

Supplementary Table 1: Detection of UCART cells in spleen of PDX-3 model mice by ddPCR.

	Treatment	CD123 expression	Days after CART injection	UCART count	ABL1 count	UCART/tABL1*10000
RNA1	Untreated	CD123+	23	3	72,642	0.4
RNA2	Untreated	CD123+	23	1	57,438	0.2
RNA3	UCART123 10M group	CD123-	188	1	70,236	0.1
RNA4	UCART123 1M group relapse	CD123-	225	13	68,332	1.9
RNA5	UCART123 10M group	CD123-	225	575	67,144	85.6
RNA10	UCART123 1M group	CD123+	119	857	42,772	200.4
RNA13	UCART123 1M group	CD123-	100	37	9,776	37.8
RNA16	UCART123 1M group relapse	CD123-	71	43	21,939	19.6
RNA17	UCART123 1M group relapse	CD123-	78	4	1,510*	26.5
NTC#	NA	NA	NA	1	43*	232.6

NTC: no template control. * Low ABL1 count less than 5,000.

UCART123 transcripts in spleen were evaluated with digital droplet PCR (ddPCR) at the time point of euthanasia due to sickness or disease progression. Copy numbers of UCART123 transcript relative to 1.0x 104 ABL1 transcripts (reference) were calculated.

Supplementary Table 2: The sequences used for primers

Name	Sequence
CD123_EX2_FWD	CCTGATCGCCCTGCCCTGTCTCC
CD123_EX6_REV	CGCTGGAGAGTCGAGAGATGTCATCG
CD123_EX5_FWD	CCAGTACGACCTGTACTTGAACGTTGC
CD123_EX9_REV	CCCGGGCTCTTATTTGTACTGTGTACG
CD123_EX9_FWD	CGTACACAGTACAAATAAGAGCCCGGG
CD123_EX12_3P_REV	GGCAGCTTCGGACGAAATTACACAGG

Supplementary Table 3: The information of antibodies

Reagent	Clone	Provider	Catalog numbers	Dilution
Anti-CD8-PE, human	BW135/80	Miltenyi Biotec Inc.	130-113-720	1:30
Anti-CD28 pure, human	15E8	Miltenyi Biotec Inc.	130-093-375	1:100
Anti-CD49d Pure, human	9F10	BD Biosciences	556634	1:500
Anti-CD107a-APC, human	H4A3	Miltenyi Biotec Inc.	130-119-955	1:100
Anti-CD123-APC, human	7G3	BD Biosciences	560087	1:20
Anti-CD123-FITC, human	7G3	BD Biosciences	558663	1:20
CellTrace CFSE Cell Proliferation Kit		Life Technologies	C34554	1:1000
Anti-hCD45-APC	HI30	BD Biosciences	555485	1:20
Anti-CD123-PE, human	9F5	BD Biosciences	555644	1:20
Anti-hCD56-APC	B159	BD Biosciences	555518	1:20
Anti-hCD5-FITC	UCHT2	BD Biosciences	555352	1:20
R-Phycoerythrin AffiniPure Goat Anti-Mouse IgG		Jackson Immuno Research Laboratories	115-115-164	1:200
Fixable Viability Dye eFluor® 780		Affymetrix Inc.	65-0865-18	1:1000
Anti-mCD45-APC	30-F11	Biolegend	103111	1:50
Anti-hCD45-FITC	H130	Biolegend	304038	1:20
Anti-hCD45-APC/Cyanine7	H130	Biolegend	304014	1:20
