# Supplementary material for: Targeting CD123 in blastic plasmacytoid dendritic cell neoplasm using allogeneic anti-CD123 CAR T cells
Source: Nat Commun. 2022 Apr 28;13:2228. doi: 10.1038/s41467-022-29669-8 (PMC9051102; doi:10.1038/s41467-022-29669-8)
Supplement: Supplementary file 2 — Reporting Summary [file 41467_2022_29669_MOESM2_ESM.pdf]

nature research

Corresponding author(s): Marina Konopleva

Last updated by author(s): Mar 17, 2022

Reporting Summary

Nature Research wishes to improve the reproducibility of the work that we publish. This form provides structure for consistency and transparency in reporting. For further information on Nature Research policies, see our Editorial Policies and the Editorial Policy Checklist.

Statistics

For all statistical analyses, confirm that the following items are present in the figure legend, table legend, main text, or Methods section.

n/a	Confirmed	
☐	☒	The exact sample size (*n*) for each experimental group/condition, given as a discrete number and unit of measurement
☐	☒	A statement on whether measurements were taken from distinct samples or whether the same sample was measured repeatedly
☐	☒	The statistical test(s) used AND whether they are one- or two-sided<br>*Only common tests should be described solely by name; describe more complex techniques in the Methods section.*
☐	☒	A description of all covariates tested
☒	☐	A description of any assumptions or corrections, such as tests of normality and adjustment for multiple comparisons
☐	☒	A full description of the statistical parameters including central tendency (e.g. means) or other basic estimates (e.g. regression coefficient) AND variation (e.g. standard deviation) or associated estimates of uncertainty (e.g. confidence intervals)
☐	☒	For null hypothesis testing, the test statistic (e.g. *F*, *t*, *r*) with confidence intervals, effect sizes, degrees of freedom and *P* value noted<br>*Give P values as exact values whenever suitable.*
☒	☐	For Bayesian analysis, information on the choice of priors and Markov chain Monte Carlo settings
☒	☐	For hierarchical and complex designs, identification of the appropriate level for tests and full reporting of outcomes
☒	☐	Estimates of effect sizes (e.g. Cohen's *d*, Pearson's *r*), indicating how they were calculated

Our web collection on statistics for biologists contains articles on many of the points above.

Software and code

Policy information about availability of computer code

Data collection	No software was used.
Data analysis	Statistical analyses were performed using Excel 2013, GraphPad Prism V8. Flow cytometry data were analyzed using by Flowjo V10. RNA sequencing data was analyzed by MAJIQ tool (version 1.03). aGCH assay data were analyzed by CytoSure Interpret software (version 4.8.32).

For manuscripts utilizing custom algorithms or software that are central to the research but not yet described in published literature, software must be made available to editors and reviewers. We strongly encourage code deposition in a community repository (e.g. GitHub). See the Nature Research guidelines for submitting code & software for further information.

Data

Policy information about availability of data

All manuscripts must include a data availability statement. This statement should provide the following information, where applicable:
- Accession codes, unique identifiers, or web links for publicly available datasets
- A list of figures that have associated raw data
- A description of any restrictions on data availability

The RNA sequencing data discussed in this publication have been deposited in NCBI's Gene Expression Omnibus and are accessible through GEO Series accession number GSE198471 (https://www.ncbi.nlm.nih.gov/geo/query/acc.cgi?acc= GSE198471). The data are available within the Article, Supplementary Information or Source Data file.

Field-specific reporting

Please select the one below that is the best fit for your research. If you are not sure, read the appropriate sections before making your selection.

[x] Life sciences [ ] Behavioural & social sciences [ ] Ecological, evolutionary & environmental sciences

For a reference copy of the document with all sections, see nature.com/documents/nr-reporting-summary-flat.pdf

Life sciences study design

All studies must disclose on these points even when the disclosure is negative.

Sample size	No sample size calculation was performed. Total 8 BPDCN samples were utilized available from the sample bank (rare disease). All cell culture experiments were performed in triplicates to allow for calculation of standard deviation, standard error of the mean and t-statistics for use in two-tailed Student's t-test. Animal numbers for each study type were determined by the investigators on the basis of previous our results with the standard disease models that were used or from pilot studies.
Data exclusions	No data were excluded from the analyses.
Replication	All experiments were performed in triplicates. We did not exclude any replicates. Information pertaining to the number of independent replications for each experiment are included in the figure legends.
Randomization	When circulating human tumor cells (i.e., BPDCN cells) were detected, mice were randomized into different treatment groups, with equal tumor burden distribution (average and variance).
Blinding	The investigators were blinded to group allocation during data collection and analysis.

Reporting for specific materials, systems and methods

We require information from authors about some types of materials, experimental systems and methods used in many studies. Here, indicate whether each material, system or method listed is relevant to your study. If you are not sure if a list item applies to your research, read the appropriate section before selecting a response.

Materials & experimental systems

n/a	Involved in the study	
[ ]	[x]	Antibodies
[ ]	[x]	Eukaryotic cell lines
[x]	[ ]	Palaeontology and archaeology
[ ]	[x]	Animals and other organisms
[ ]	[x]	Human research participants
[x]	[ ]	Clinical data
[x]	[ ]	Dual use research of concern

Methods

n/a	Involved in the study	
[x]	[ ]	ChIP-seq
[ ]	[x]	Flow cytometry
[x]	[ ]	MRI-based neuroimaging

Antibodies

Antibodies used	Anti-CD8-PE, human from Miltenyi Biotec Inc. 130-113-720. Dilution 1:30<br>Anti-CD28 pure, human from Miltenyi Biotec Inc. 130-093-375. Dilution 1:100<br>Anti-CD49d Pure 9F10, human from BD Biosciences 556634. Dilution 1:500<br>Anti-CD107a-APC, human from Miltenyi Biotec Inc. 130-119-955. Dilution 1:500<br>Anti-CD123-APC 7G3, human from BD Biosciences 560087. Dilution 1:20<br>Anti-CD123-FITC 7G3, human from BD Biosciences 558663. Dilution 1:20<br>CellTrace CFSE Cell Proliferation Kit from Life Technologies C34554. Dilution 1:1000<br>Anti-hCD45 antibody from BD Biosciences 555485. Dilution 1:20<br>Anti-CD123-PE, human from BD Biosciences 555644. Dilution 1:20<br>Anti-hCD56-APC antibody from BD Biosciences 555518. Dilution 1:20<br>Anti-hCD5-FITC antibody from BD Biosciences 555352. Dilution 1:20<br>R-Phycoerythrin AffiniPure Goat Anti-Mouse IgG from Jackson ImmunoResearch Laboratories, Inc. 115-115-164. Dilution 1:200<br>Fixable Viability Dye eFluor® 780 from Affymetrix Inc. 65-0865-18. Dilution 1:1000<br>Anti-mCD45-APC from Biolegend 103111. Dilution 1:50<br>Anti-hCD45-FITC from Biolegend 304038. Dilution 1:20<br>Anti-hCD45-APC/Cyanine7 from Biolegend 304014. Dilution 1:20
Validation	All the antibodies used in this work have been validated by the companies where these antibodies were purchased from.

Eukaryotic cell lines

Policy information about cell lines

Cell line source(s)	The BPDCN CAL-1 cells were obtained from Takahiro Maeda (Nagasaki University). MOLM13 acute myeloid leukemia (AML) and Jurkat T cell leukemia cells were obtained from ATCC (Manassas, VA) or Deutsche Sammlung von Mikroorganismen und Zellkulturen (Braunschweig, Germany).
Authentication	All cells were validated by short-tandem repeat profiling prior to their use in these experiments
Mycoplasma contamination	All cell lines used in these studies were tested for mycoplasma every six months. All cell lines used in these studies were mycoplasma negative.
Commonly misidentified lines (See ICLAC register)	No commonly misidentified cell lines were used in this study.

Animals and other organisms

Policy information about studies involving animals; ARRIVE guidelines recommended for reporting animal research

Laboratory animals	NOD scid gamma (NSG) mice and NSGS mice (NOD-scid IL2Rgnull-3/GM/SF, NSG-SGM3) were used in this study. The mice were female, 8-12 weeks old. Animals were maintained in rooms under controlled conditions of temperature, humidity, photoperiod (12h light/12h dark) and air exchange. Animals were maintained in SPF conditions. Room temperature (22 ± 2°C) and humidity (55 ± 10%) were continuously monitored.
Wild animals	The study did not involve wild animals.
Field-collected samples	No field collected samples were used in the study.
Ethics oversight	The animal study was approved by the Animal Care and Use Protocol at The University of Texas MD Anderson Cancer Center (Immunotherapy approaches in leukemia, 00001446-RN00) and Institutional Review Board approved protocol (PA14-0157, A Study to Evaluate Patient Samples with Blastic Plasma Cytoid Dendritic Cell Neoplasm)

Note that full information on the approval of the study protocol must also be provided in the manuscript.

Human research participants

Policy information about studies involving human research participants

Population characteristics	De-identified diagnosis-obtained cryopreserved primary BPDCN cells were obtained from 8 patients through The University of Texas MD Anderson Cancer Center. Table 1 showes the characteristics of primary BPDCN patients' samples.
Recruitment	Primary BPDCN cells and AML cells were collected from patients who had consented to research protocols approved by the Institutional Review Board at The University of Texas MD Anderson Cancer Center for analysis of hematologic malignancies.
Ethics oversight	Primary BPDCN cells and AML cells were collected from patients who had consented to research protocols approved by the Institutional Review Board at The University of Texas MD Anderson Cancer Center (PA14-0157, A Study to Evaluate Patient Samples with Blastic Plasma Cytoid Dendritic Cell Neoplasm). This animal study was performing under Animal Care and Use Protocol (Immunotherapy approaches in leukemia, 00001446-RN00). The production of R&D grade batches of UCART123 was done using commercially available NHPBMC cells ordered from ALLCELLS or HEMACARE. These are normal human peripheral blood mononuclear cells derived from an apheresis collection. Donors have been screened for viral status and the collection protocols and donor-informed consent are approved by an Institutional Review Board (IRB), with strict oversight. HIPAA compliance and approved protocols are also followed. The commercially available NHPBMC cells used to generate the UCART cells described in the manuscript are for Research Purposes Only and were used according to local licenses and regulations (French Ministry of Higher Education, Research and Innovation – French Biotechnology Council).

Note that full information on the approval of the study protocol must also be provided in the manuscript.

Flow Cytometry

Plots

Confirm that:

- [x] The axis labels state the marker and fluorochrome used (e.g. CD4-FITC).
- [x] The axis scales are clearly visible. Include numbers along axes only for bottom left plot of group (a 'group' is an analysis of identical markers).
- [x] All plots are contour plots with outliers or pseudocolor plots.
- [x] A numerical value for number of cells or percentage (with statistics) is provided.

Methodology

Sample preparation	Primary BPDCN cells and AML cells were collected from patients who had consented to research protocols approved by the Institutional Review Board at The University of Texas MD Anderson Cancer Center for analysis of hematologic malignancies (PA14-0157, A Study to Evaluate Patient Samples with Blastic Plasma Cytoid Dendritic Cell Neoplasm). Bone marrow aspirate mononuclear cells from patients with BPDCN were purified by Ficoll density centrifugation using standard procedures. Lymphocytes were isolated by using Lymphocyte Separation Medium. Cells were frozen in FBS and dimethyl sulfoxide and stored in liquid nitrogen until use.
Instrument	Gallios 10 Colors, 3 Lasers (A94303) from Beckman Coulter was used for flow cytometry.
Software	Data were analyzed using FlowJo software
Cell population abundance	The acquisition was stopped after 5,000 DAPI- viable cells (if achievable) for each sample. All the events were saved during acquisition.
Gating strategy	We used the negative control for the gating strategy of positive and negative cell population.

☒ Tick this box to confirm that a figure exemplifying the gating strategy is provided in the Supplementary Information.
